# Accurate species identification of food-contaminating beetles with quality-improved elytral images and deep learning

**DOI:** 10.3389/frai.2022.952424

**Published:** 2022-08-12

**Authors:** Halil Bisgin, Tanmay Bera, Leihong Wu, Hongjian Ding, Neslihan Bisgin, Zhichao Liu, Monica Pava-Ripoll, Amy Barnes, James F. Campbell, Himansi Vyas, Cesare Furlanello, Weida Tong, Joshua Xu

**Affiliations:** ^1^Department of Mathematics and Applied Sciences, University of Michigan-Flint, Flint, MI, United States; ^2^Division of Bioinformatics and Biostatistics, National Center for Toxicological Research, US Food and Drug Administration, Jefferson, AR, United States; ^3^Food Chemistry Lab 1, Arkansas Regional Laboratory, Office of Regulatory Affairs, US Food and Drug Administration, Jefferson, AR, United States; ^4^Office for Food Safety, Center for Food Safety and Applied Nutrition, US Food and Drug Administration, College Park, MD, United States; ^5^Stored Product Insect and Engineering Research Unit, US Department of Agriculture, Manhattan, KS, United States; ^6^HK3 Lab, Milan, Italy

**Keywords:** food-contaminating beetle, species identification, deep learning, convolutional neural networks, machine learning, food safety, image classification

## Abstract

Food samples are routinely screened for food-contaminating beetles (i.e., pantry beetles) due to their adverse impact on the economy, environment, public health and safety. If found, their remains are subsequently analyzed to identify the species responsible for the contamination; each species poses different levels of risk, requiring different regulatory and management steps. At present, this identification is done through manual microscopic examination since each species of beetle has a unique pattern on its elytra (hardened forewing). Our study sought to automate the pattern recognition process through machine learning. Such automation will enable more efficient identification of pantry beetle species and could potentially be scaled up and implemented across various analysis centers in a consistent manner. In our earlier studies, we demonstrated that automated species identification of pantry beetles is feasible through elytral pattern recognition. Due to poor image quality, however, we failed to achieve prediction accuracies of more than 80%. Subsequently, we modified the traditional imaging technique, allowing us to acquire high-quality elytral images. In this study, we explored whether high-quality elytral images can truly achieve near-perfect prediction accuracies for 27 different species of pantry beetles. To test this hypothesis, we developed a convolutional neural network (CNN) model and compared performance between two different image sets for various pantry beetles. Our study indicates improved image quality indeed leads to better prediction accuracy; however, it was not the only requirement for achieving good accuracy. Also required are many high-quality images, especially for species with a high number of variations in their elytral patterns. The current study provided a direction toward achieving our ultimate goal of automated species identification through elytral pattern recognition.

## Introduction

A large group of nuisance insects that contaminate grains and other food items are commonly termed pantry beetles (Bell, 2013). They are notorious for spoiling stored grain and processed food products, leading to significant economic damage (Belluco et al., 2013). Some of these pantry beetles are aggressively invasive and can cause damage to local agriculture and ecological insects if they spread through the transportation of contaminated food products (Heeps, 2016). Some of the pests also pose a serious threat to public health, as they are active carriers of pathogens (Olsen et al., 2001).

To counter such adversities, food grains and products are monitored and routinely screened for pantry beetles or their remains (Bell, 2013; Belluco et al., 2013). The most common and widely-used method involves highly-trained analysts manually screening food samples for insect remains using optical microscopes. Any insect or insect remains found are then scrutinized using a comparison optical microscope to match the patterns from the insect fragments with reference images to identify the exact insect species, genus, or family. This identification step is crucial, as each species poses different threat levels and their contamination may require different methods of management and regulatory procedures. Currently, no reliable alternatives to the manual screening method are available, as spectroscopic or PCR-based detection techniques have remained challenging for this application. Moreover, due to the manual nature of the microanalysis, the current method is highly dependent on the experience and expertise of the individual analyst, making it more susceptible to human error and higher variation across institutions. Also, manual methods are difficult to scale up, hindering the screening of a larger number of samples in a shorter time frame, especially in the absence of experienced and dexterous analysts.

Species identification through image analysis has been explored for efficient taxonomical and environmental applications for several years (Norouzzadeh et al., 2018; Terry et al., 2019; Høye et al., 2020). These computer-aided applications have tried to address a wide range of problems from food safety to identification of insect pests (Daly et al., 1982; Weeks et al., 1997; O'Neill et al., 2000; Larios et al., 2008; Yalcin, 2015). With the advent of machine learning methods, image-based species identification has gained further momentum and well-known discriminative models such as support vector machines (SVM) (Cortes and Vapnik, 1995) and generative models have been widely adopted for insect classification (Martineau et al., 2017). Examples of these models include, but are not limited to: insect or pest identification using SVM (Qing et al., 2012; Wang et al., 2012; Yang et al., 2015), honeybee and moth identification with decision trees (Mayo and Watson, 2007; da Silva et al., 2015), and red palm weevil and insect recognition systems through neural networks (Al-Saqer and Hassan, 2011; Wang et al., 2012). With increasing computational power, more complex neural network architectures, i.e., deep learning (DL) approaches have recently helped in tackling more challenging tasks in the field of food and agricultural science (Lee et al., 2015; DeChant et al., 2017; Lu et al., 2017; Zhang et al., 2018). Although there have been relatively fewer DL studies to identify filth elements for food contamination (Reinholds et al., 2015; Bansal et al., 2017), variations of DL designs such as Region-based Fully Convolutional Network (R-FCN), convolutional block attention module (CBAM), convolutional neural network (CNN) and pre-trained models have shown promising performances for pest, stored-grain insect, and fly classification (Chen et al., 2020; Kuzuhara et al., 2020; Shi et al., 2020). The DL models have not only achieved high classification accuracies, but also offered a new way of feature extraction embedded in the process as an alternative to conventional features such as domain-dependent, global, local, and mid-level features (Martineau et al., 2017).

We have also investigated similar approaches, i.e., machine learning techniques, with the aim of automating the identification process of pantry beetles whose elytra (hardened forewing) have unique patterns that can be considered as fingerprints or features. In a previous study, we demonstrated that a specific pantry beetle species could indeed be identified through elytral pattern recognition using machine learning (Martin et al., 2016). In our subsequent study, we observed that classical machine learning techniques such as artificial neural network (ANN) and SVM could be used for this application (Bisgin et al., 2018). However, optimized ANN and SVM models yielded about 80 and 85% of average accuracies, respectively. We observed that some species consistently performed less than others; which could be attributed to their misidentification with another species from the same genus or family with similar or near-identical elytral patterns. We further studied more advanced machine learning techniques such as CNN, which also performed similarly (Wu et al., 2019).

Our findings in our earlier studies led us to scrutinize the image set and observe that images lacking visual clarity due to the reflective glare of the elytra surface were more prone to misidentification. To remedy this, we amended the optical and imaging settings and optimized the imaging conditions to obtain a high-quality image set unaffected by artifacts and showing the finer details of an elytron (Bera et al., 2021). We hypothesized that using such a high-quality image set would help us achieve a near-perfect prediction accuracy in identifying each pantry beetle species. In the current study, therefore, we tested this hypothesis by using a CNN model on an extended dataset which consisted of high-quality images of 27 species. We further shed light on the impact of enhanced images of 12 species in the same dataset that were previously studied. Our experiments showed both the utility of the prediction framework and the improvement in species identification due to image quality which could potentially guide any future efforts for auto-detection tools.

The rest of the paper is organized as follows: Section Material and methods details the dataset for 27 species and introduces our approach, Section Results and discussion presents our results, Section Discussion discusses our findings, and Section Conclusion concludes our work.

## Materials and methods

### Beetle sample collection and image acquisition

We elaborated on the details of sample collection, preparation, and imaging technique in our previous publications on imaging optimization (Bisgin et al., 2018; Wu et al., 2019; Bera et al., 2021). Briefly, we used 12 different pantry beetle species harvested from our in-house collection. We chose these species due to their prevalence and significance in food contamination, especially in North American food samples. Another 15 different species were collected from the U.S. Department of Agriculture's (USDA) Animal and Plant Health Inspection Service (APHIS) laboratory. Elytra from each beetle specimen were harvested, thoroughly cleaned through sonication in an ethanol solution, and subsequently preserved in 70% ethanol prior to imaging. Table 1 shows the full list of 27 species which include both our in-house collection (12 species) and additional 15 species.

**Table 1:** The complete list of pantry beetles used in this study, listed alphabetically by their family, genus, species and common names, with abbreviations.

	**Family**	**Genus**	**Species**	**Common Name**	**SP Id new**
1	Anthribidae	*Araecerus*	*fasciculatus*	Coffee Bean Weevil	AAF
2	Anobiidae	*Lasioderma*	*serricorne*	Cigarette Beetle	ALS
3	Anobiidae	*Stegobium*	*paniceum*	Drugstore Beetle	ASP
4	Bostrichidae	*Rhyzopthera*	*dominica*	Lesser Grain Borer	BRD
5	Chrysomelidae	*Callosobruchus*	*maculatus*	Cowpea Weevil	CCM
6	Curculionidae	*Sitophilus*	*granarius*	Granary Weevil	CSG
7	Curculionidae	*Sitophilus*	*oryzae*	Rice Weevil	CSO
8	Curculionidae	*Sitophilus*	*zeamaise*	Maize Weevil	CSZ
9	Dermestidae	*Attagenus*	*Unicolor*	Black Carpet Beetle	DAU
10	Dermestidae	*Trogoderma*	*inclusum*	Cabinet Beetle	DTI
11	Laemophloeidae	*Cryptolestes*	*ferrugineus*	Rusty Grain Beetle	LCF
12	Laemophloeidae	*Cryptolestes*	*pusillus*	Flat Grain Beetle	LCP
13	Laemophloeidae	*Cryptolestes*	*turcicus*	Flour Mill Beetle	LCT
14	Silvanidae	*Ahasverus*	*advena*	Foreign Grain Beetle	SAA
15	Silvanidae	*Ahasverus*	*species*	Fungus Beetle	SAS
16	Silvanidae	*Cathartus*	*quadricollis*	Squarenecked Grain Beetle	SCQ
17	Silvanidae	*Oryzaephilus*	*mercator*	Merchant Grain Beetle	SOM
18	Silvanidae	*Oryzaephilus*	*surinamensis*	Saw-toothed Grain Beetle	SOS
19	Tenebrionidae	*Cynaeus*	*angustus*	Larger Black Flour Beetle	TCA
20	Tenebrionidae	*Gnatocerus*	*cornutus*	Broad-horned Flour Beetle	TGC
21	Tenebrionidae	*Latheticus*	*oryzae*	Longheaded Flour Beetle	TLO
22	Tenebrionidae	*Lophocateres*	*pusillus*	Siamese Grain Beetle	TLP
23	Tenebrionidae	*Palorus*	*ratzeburgii*	Smalleyed Flour Beetle	TPR
24	Tenebrionidae	*Tribolium*	*castaneum*	Red Flour Beetle	TTCa
25	Tenebrionidae	*Tribolium*	*confusum*	Confused Flour Beetle	TTCo
26	Tenebrionidae	*Tribolium*	*Destructor*	Dark Flour Beetle	TTD
27	Tenebrionidae	*Tribolium*	*madens*	Black Flour Beetle	TTM

The harvested elytra were then air-dried and imaged using stereo microscopes (Leica M205, Allendale, New Jersey). Unlike the older image set, which was subjected to varied magnification (in the 75–100× range) and two-point reflected light, we used a fixed magnification of 100× and transmitted light for this study. These amendments significantly reduced glare spots and other imaging artifacts, and drastically improved the clarity of elytral patterns (Bera et al., 2021). We used a Leica MC170HD camera to acquire the images with an image resolution set to 2,592 × 1,944 dpi (dots per inch, the highest resolution available). In this study, only images from the ventral side (underside) of the elytra were used. The concave shape of the elytra naturally preserves the ventral side elytral patterns. This selection allowed us to focus our attention on only the pattern recognition aspect without having to worry about such artifacts as variation or loss of setae (surface hair) or other sample damages that often occur on the frontal side of the elytra during food or sample preparation steps.

We used 20 elytral images per species. Each image subsequently was divided into smaller subimages (tiles) to simulate physical fragmentation of the elytra that are often observed in contaminated samples. This simulated fragmentation step was critical to our application, as it allowed us to increase the sample size and to validate our algorithms in close to real-life scenarios, in which elytral fragments are the only viable remains found in contaminated food samples.

### Image preprocessing

Each image frame (captured at 100× magnification) had the elytra at the center of the white background. Thus, in the first step of preprocessing, we removed the white background by determining the elytral border (line of maximum change in contrast). Next, we randomly split images belonging to the same species to construct training and test sets by observing a 4:1 ratio, as shown schematically in Supplementary Figure 1, which was the same practice we used in our previous studies. Since an early study showed the utility of images with a size of 448 × 448 (Wu et al., 2019), we randomly cropped 100 regions so that each image was the same size. These sub-images guaranteed they would be inside the borders detected in the previous step and allowed to have overlap. This resulted in 46,300 training and 10,800 test images. By following such an exercise, we ensured that all sub-images of a particular image were put either in the training or test set in order to prevent information leak. This “blind” cross-validation strategy reduced bias and minimized the possibility of overfitting.

### Convolutional neural network and the model structure

For the classification task here, we adopted CNNs, which have been widely used in the research community for image classification and segmentation in recent years (Lawrence et al., 1997; Krizhevsky et al., 2012; LeCun, 2021). The ability of CNN to learn features while applying convolutional filters during the training stage makes it appealing and different from conventional image classification methods (Zheng et al., 2006). These types of deep neural network structures comprise cascaded convolutional and pooling layers in which filters are utilized to attain the most informative features that eventually provide significantly reduced image sizes. The CNN final output is then passed to a dense layer in a flattened representation, allowing passage to subsequent dense layers that finally terminate in another fully-connected layer with a number of neurons equal to the number of classes (i.e., species, in our case).

**Figure 1:**
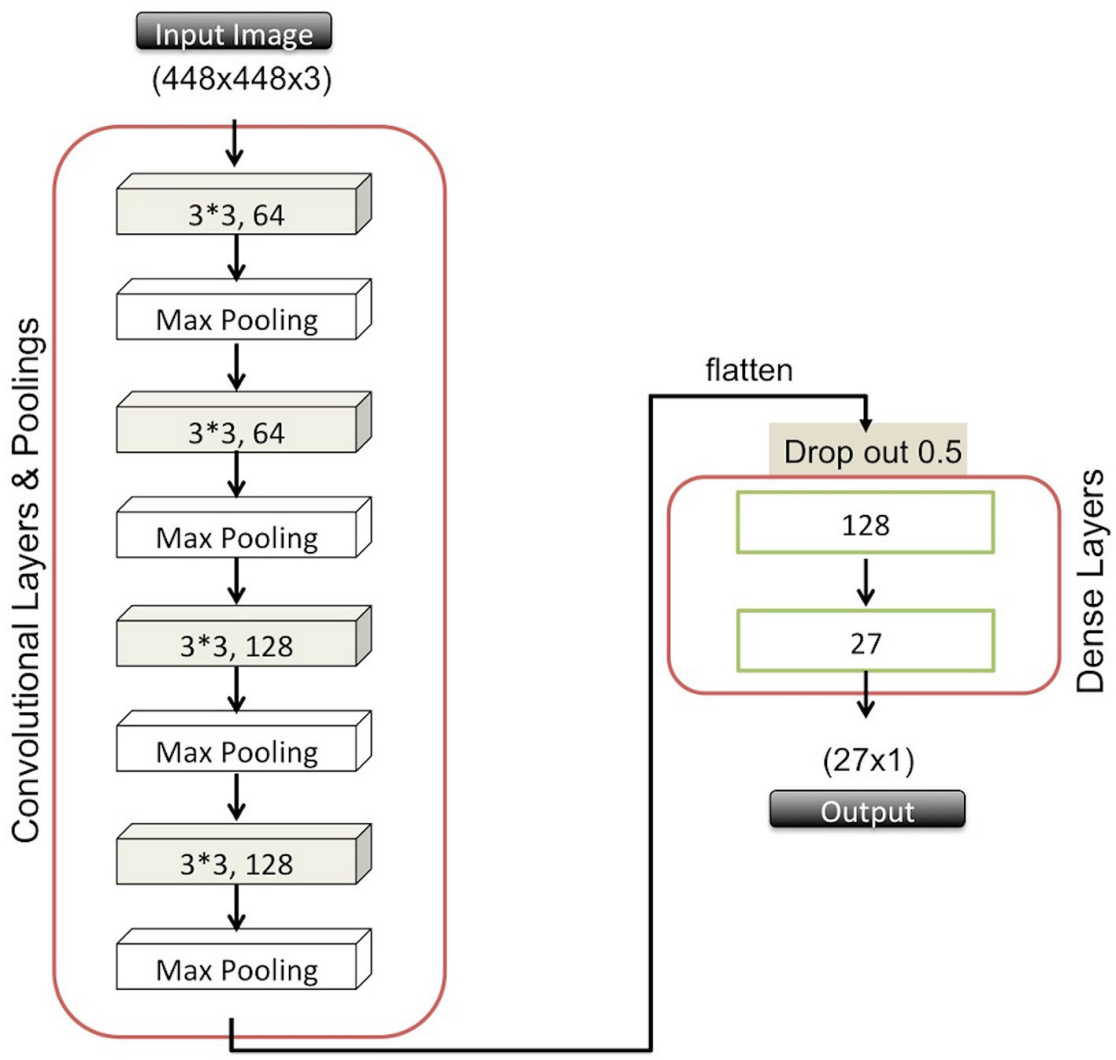
Overview of the CNN architecture.

We constructed a CNN by using Keras (Chollet, 2015), which is an application programming interface (API) that runs the Tensorflow machine learning platform (Abadi et al., 2016) in the backend and offers further image preprocessing utilities for more generalizable models. Specifically, our network architecture consists of four convolutional layers along with corresponding pooling layers. These perform downsampling, usually by either choosing the maximum or average value in a given region, and two additional dense layers. We employed 3 × 3 filters in the convolutional layers that were followed by max pooling layers using 2 × 2 windows to choose the maximum value. In order to avoid overfitting, we further adapted the dropout approach that randomly ignores some units at a desired level to prevent coadapting (Srivastava et al., 2014). In Figure 1, we illustrate the details of our network structure, listing all six layers and the number of nodes for each layer. We used Rectified Linear Unit (ReLU) activation function in the first five layers. In the final layer, we used a softmax function due to the multi-class nature of our predictions. For the optimizer, we used the Adam algorithm because of its efficient management of larger datasets and parameters (Kingma and Ba, 2014).

Keras's data augmentation features enabled us to artificially increase the sample size (i.e., number of subimages). Additionally, it helped generalize the model by applying image processing functions to the existing training samples. These functions perform image manipulations, such as rotations, that lead to a more diverse and larger set of images derived from the original set. We list details about the augmentation options and parameter values used in our study in Table 2. From the details shown in Table 2, we derived an augmented training set which could include additional images that might be shifted 20%, rotated 30 degrees, magnified 15%, sheared 10%, and horizontally flipped. If any pixels were lost due to the operations and needed to be filled to keep the image integrity, the nearest pixels could be used.

**Table 2:** List of augmentation options and parameter values used in our study.

**Option**	**Explanation**	**Value**
rotation_range	Creates images with random rotations up to N degrees.	40
width_shift_range	Handles off-center objects by artificially creating shifted versions of the training data	0.2
height_shift_range		0.2
shear_range	Shear angle in counterclockwise direction in degrees	0.2
zoom_range	Random zoom range	0.2
horizontal_flip	Creates random flips of the image (supposes you feed a mirror image)	True
fill_mode	Helps in filling values outside the boundaries of an image	nearest

### Model training and validation

Keras offers a user-friendly interface for data augmentation and experimental design, including the arrangement of training and test sets consisting of image folders maintained by the *ImageDataGenerator* module of the *keras_preprocessing* library. In our case, for 27 species we created a training directory that included 27 folders, from which class labels were inherited. Similarly, we created a validation directory using the *flow_from_directory* function.

We passed these settings to the *fit_generator* function, along with the compiled neural network detailed above, with the *categorical_crossentropy* loss function, *adam* optimizer, and the default batch size (Bisgin et al., 2018). We trained our model for 100 epochs and tested its performance on the validation images after each epoch.

### Model evaluation

As in our previous studies, we first computed the accuracy values for each species by computing the mean and standard deviation for each round of validation (Bisgin et al., 2018; Wu et al., 2019). This yielded a confusion matrix after the cross-validation from which true positive (TP), false positive (FP), true negative (TN), and false negative (FN) were computed. These were subsequently used to calculate the prediction parameters, namely Precision, Sensitivity (Recall), Specificity, Matthews Correlation Coefficient (MCC) using the standard formula, which can also be found in our previous report (Bisgin et al., 2018). Average prediction accuracy was also calculated by averaging species-wise accuracies.

### Code and experimental environment

Given the significantly increased image size (average 14 mb per full elytra image and 600 kb per sub-images), we used the NCTR/FDA High-Performance Computing Cluster containing approximately 1100 CPU cores. The script used in this study can be found in github^1^.

## Results

### Beetle species and the classifier

We initiated the study with 15 species of food-contaminating beetles most prevalent to North America. In the later part of the study, this number was expanded to 27 species. Table 1 contains a list of test species alphabetized by their family names with details on their nomenclature; namely, family, genus, species, and common names, along with their abbreviations. Those abbreviations were used to refer to each tested species. Supplementary Figure 2 shows some of the representative elytra images. For comparison, we provided images obtained though both the traditional and optimized methods. It was quite evident that imaging optimization significantly improved image quality and clarity of the elytral patterns. Compared to the traditionally acquired image set, the optimized image set was devoid of such artifacts as glare spots and other surface anomalies. Details on the imaging improvements, described elsewhere, are beyond the scope of this discussion (Bera et al., 2021). This image set subsequently was processed to obtain the set of sub-images used for our model.

### Model summary

**Figure 2:**
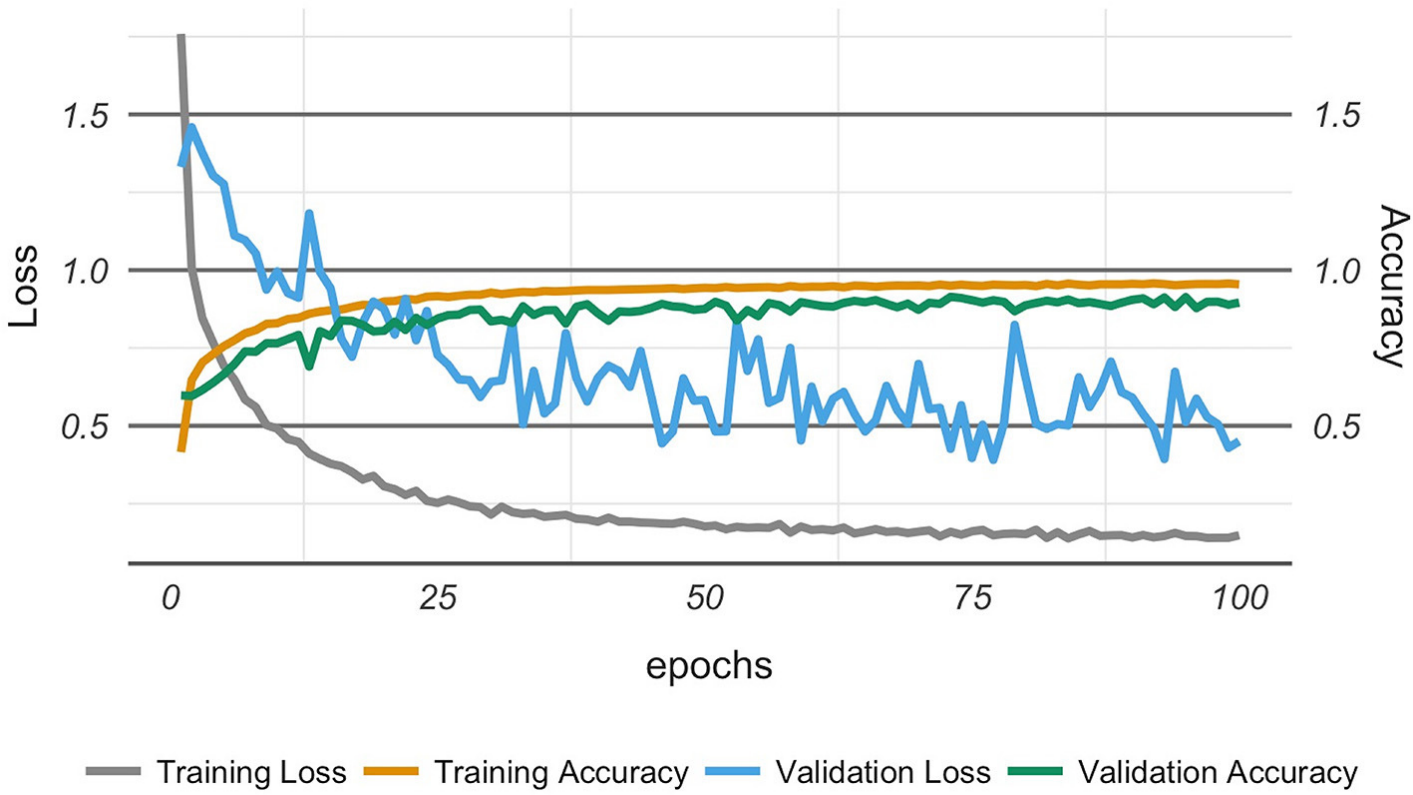
Model optimization showing the model achieving optimal performance after about 50 epochs.

The analysis of training and validation progress of the 27 classes along epochs is reported in Figure 2. We observed that the training loss (i.e., categorical cross-entropy) began to stabilize after ~50 epochs, beyond which the decrease was much more gradual. Also, we observed that testing accuracy approached saturation after ~50 epochs. Both observations might indicate that the model had reached nearly optimal accuracy, and that 50 epochs would have been enough, which was close to our earlier observations. However, the loss function for the testing (validation loss) fluctuated, but tended to stay in a limited bandwidth around the value at 50 epochs.

### Species-wise performance and comparison

**Figure 3:**
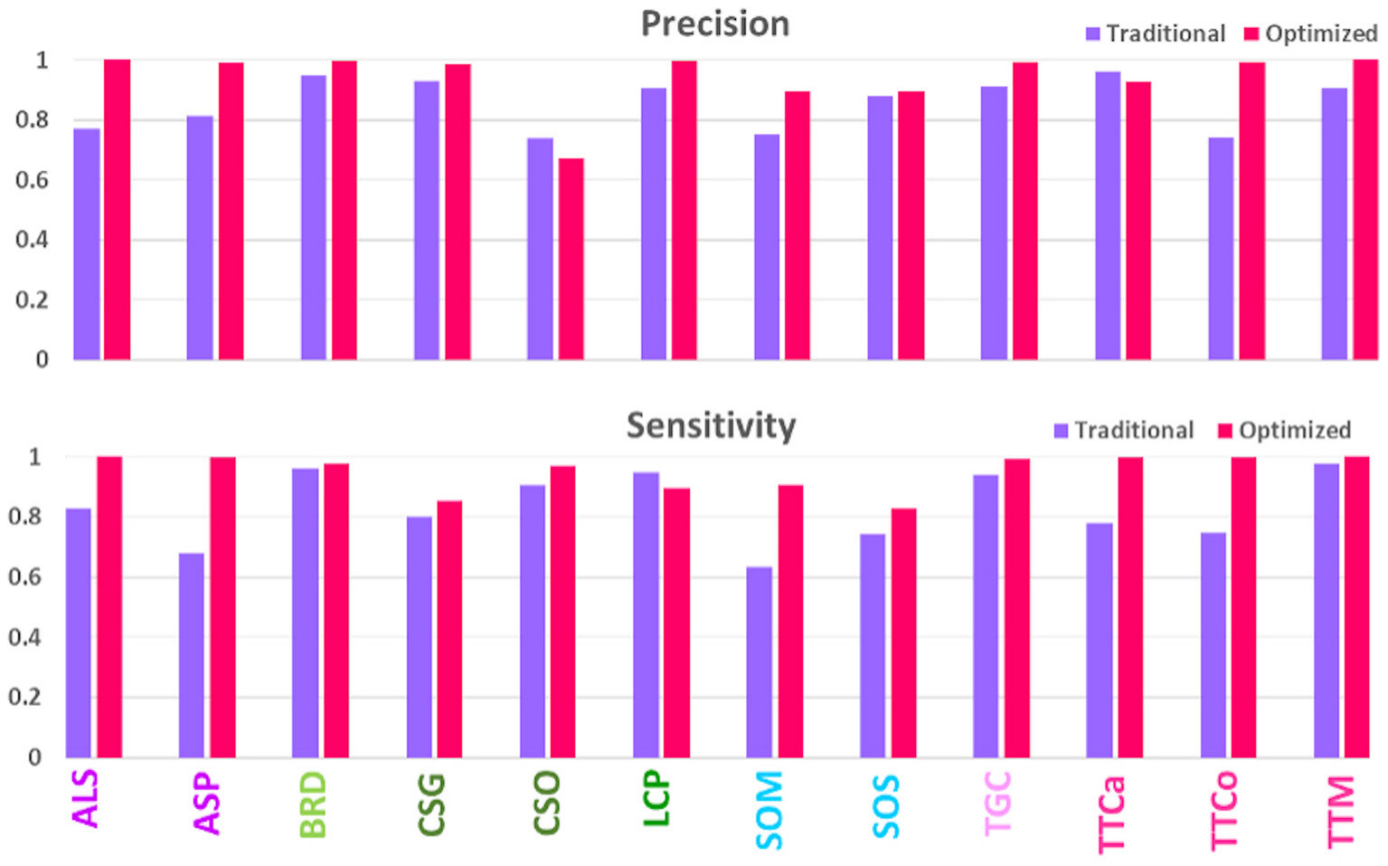
Comparison of model performances on validation sets of traditionally- and optimally-acquired images.

To test the hypothesis that a high-quality image set may increase prediction accuracy, we made a head-to-head comparison of the prediction results (Recall and Precision) for the same 12 species for earlier and current image sets, as shown in Figure 3. Evidently, the newer high-quality image set improves the prediction performance for most species, with an average prediction accuracy increasing from 80% to above 90%. The improvements were particularly notable for such species as ALS and ASP, SOM and SOS, and TTCa and TTCo; these had previously been difficult to accurately identify, however, can now be identified with >90% accuracy. These 12 species, especially, SOM, SOS, TTCa and TTCo, are some of most commonly encountered pantry beetles in North America. Therefore, improving the accuracy of their prediction identification will have regulatory significance. The traditionally-obtained images with higher artifacts and lower quality lacked the pattern clarity to distinguish one species from another. This was particularly true for species with near-identical elytral patterns (due to their genetic similarity) and belonging to the same genus and/or family [referred to as “difficult pairs” in our previous works (Bisgin et al., 2018; Wu et al., 2019)]. The high-quality images significantly improved the pattern clarity, allowing for distinct identification of each species, even within the difficult pairs. To our surprise, we observed exceptions to this general trend, especially for the species CSO. Of all 12 species, this one performed the poorest and showed a significant decrease in prediction accuracy compared to the traditionally-acquired images. The image set for this particular species possibly contained an anomaly, resulting in this decrease.

### Expansion to more species—performance parameters

**Figure 4:**
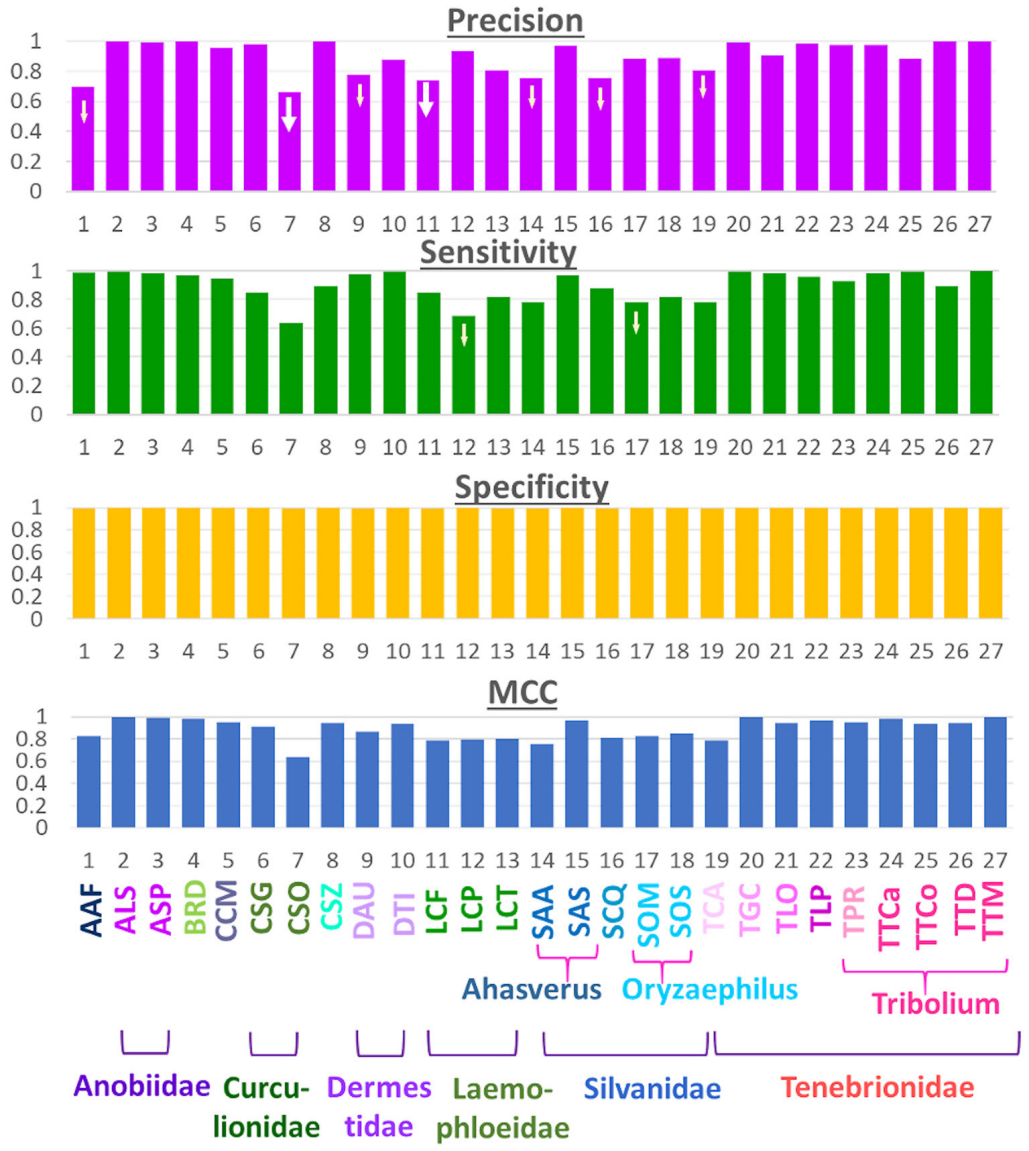
Performance metrics for the 27-class model.

Expanding the number of species to 27 enabled us to verify the observations made with the initial 12 species with our newly built model in this study. Four prediction parameters, namely Precision, Recall (or Sensitivity), Specificity and MCC, for these species are presented in Figure 4. The general trend of improved prediction is evident from this figure. Specificity values for all the species validate our hypothesis that high-quality images can improve prediction accuracy. However, there were exceptions to the general trend; as some species, such as CSO and LCP, performed quite poorly. Several other species, namely AAF, CSG, LCF, SAA, SOM, SOS, and TCA, performed below average, i.e., 90%. This suggests poor performance is not a singular anomaly in the image set of one species. Instead, there may be underlying factors that play a crucial role in a species' prediction performance and these need further research. One possibility, as we observed previously, is that species with similar elytral patterns (belonging to the same genus and/or family) were confused with one another during the prediction.

### Confusion matrix

**Figure 5:**
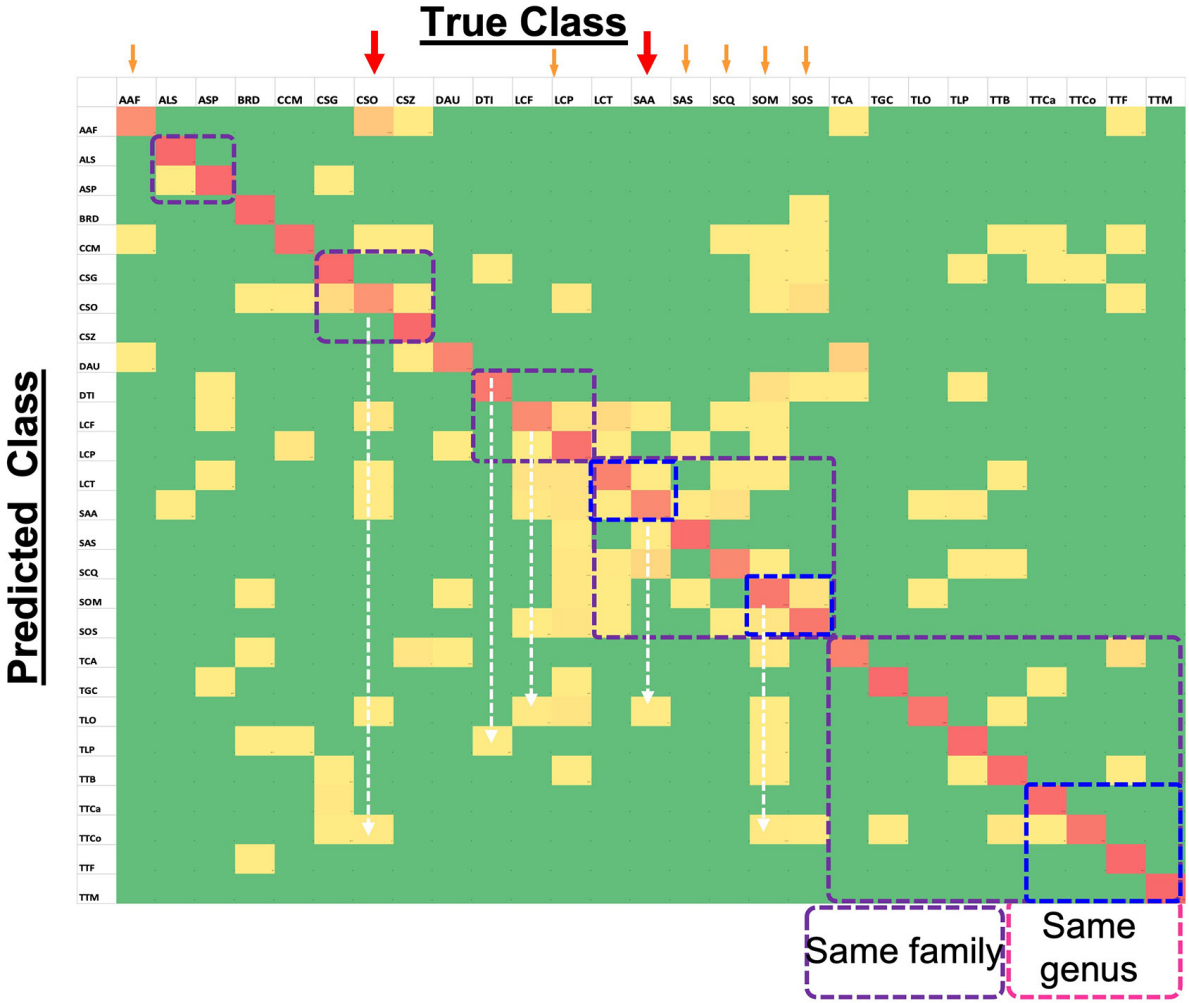
Confusion Matrix for 27-class task (computed on test set) showing the level of agreement between true and predicted classes. Red colored tiles (diagonal) represent correct classification of each species and represent values between 67% and 100%. Yellow tiles represent incorrect classification ratios that are non-zero and go up to 28%. Finally, green tiles represent zero values which means targeted species is not confused with the corresponding species.

Figure 5 shows the confusion matrix for all the species, with horizontal rows showing the True class and vertical rows the Predicted class. It is evident that overall performance of the model is quite accurate, as the red diagonal entities are clearly prominent. Although the model is far from perfect, as one can observe several non-diagonal entities in yellow, it is a good working model since the deviations were fairly low (mostly yellow and not orange non-diagonal entities) as indicated by the color scale. A closer look at the matrix, especially for the poorly-performing species (marked with red arrows) such as CSO, indicated that its low prediction performance was not due to the similarity of elytral patterns with a species from the same genus or family (marked by dotted squares). Rather, it was being predicted for several different species across various families. For instance TTCo was predicted as SOM and SOS for 9 and 6 times, respectively, compared to TGC, which is in the same family. This suggests that the image quality that showed distinction (or resulted in confusion) between similar elytral patterns is not the major factor at play on our data. We made a similar observation for the second-lowest performer, LCF, which was also predicted beyond its own genus and/or family. Other low-performing species, such as AAF, SAA, SCQ, SOM, SOS, and TCA, showed comparable trends. The two-dimensional UMAP representation of all classes based on their extracted 128 features from the last layer of the network (Supplementary Figure 3) also illustrates misclassified species. This observation further bolstered our speculation that something other than pattern clarity may be affecting the prediction performance, and deserved detailed discussion.

## Discussion

In most academic and research settings, the architecture of the model often receives more attention than does the quality of the data, possibly because cleaning the dataset often is beyond the scope of many researchers. This has been found to be true, particularly in image classification for species identification applications. Users of prediction models, even models with the best-known architecture, have found achieving good accuracy for noisy datasets challenging as quality of the data has impact on the classifier performance (Sáez et al., 2016). Our study also highlights this fact in the context of species identification and food safety, as the prediction performance showed improvement when a better-quality dataset was used to build the model.

**Figure 6:**
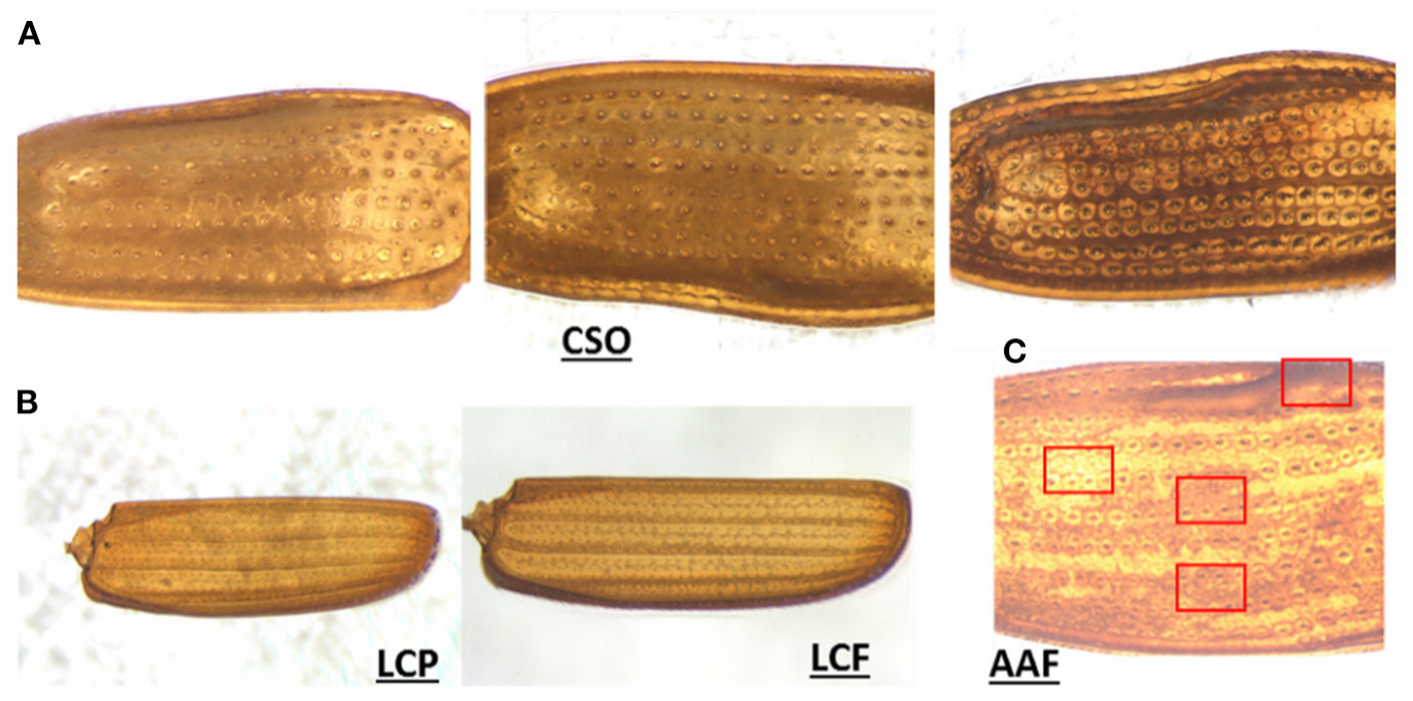
Representative images of elytral variation. **(A)** Intraspecies pattern variation in CSO (possibly due to the difference in maturity), **(B)** pattern variation due to background interference in LCP and LCF, and **(C)** regional variation in elytral patterns in AAF.

Furthermore, our results indicated the importance and relevance of other factors beyond data quality. As discussed previously, we observed that species performing below average were not being inaccurately predicted or confused with another species from their own genus and/or family due to elytral pattern similarity, but were being misclassified into various different and unrelated species. To better understand this problem, we delved deeper and looked through the images of those species. Figure 6A shows three different elytral images of the same species, CSO. The difference in elytral patterns are obvious, and believed to be mostly due to age of the beetle. However, differences could also be due to sex and/or individual variation, as the older beetles tend to develop a darker elytral color and prominent pattern, possibly to attract a mate. These variations are not uncommon and were found in such other species as SAA and SOS (Supplementary Figure 4), which also performed poorly.

Surprisingly, these species did not perform so poorly in our previous models using ANN and SVM. Supplementary Figure 5 shows the Recall and Precision comparisons for ANN, SVM, and CNN models using a conventionally-obtained (lower quality) image set than that of the present model (CNN, using a higher-quality image set). In comparison to ANN and SVM models, the performances of CSO dropped in both CNN models (using conventional and high-quality image sets), even though the CNN model is known to, in most cases, outperform ANN and SVM (Shin and Balasingham, 2017; Senyurek et al., 2019). We argue that this anomaly is due to the difference between explicitly defining features or trusting the CNN to develop its own feature extraction internally. In both ANN and SVM, the image features (such as size, shape, distribution, and color of the elytral pattern), were preprocessed before being used for training and testing the model. It is during this feature selection process that the intraspecies variations in elytral patterns probably did not get selected in the top-ranked features, as they appeared in only a handful of species. Subsequently, they remained unused in the ANN and SVM models and showed no influence in performance. On the other hand, output of convolutional layers served as the feature set in the current model, which could not take advantage of earlier select features, possibly causing a decrease in performance.

Unlike CSO, the species LCP (the second-lowest performer) did not show significant intraspecies variation. On minute observation, we found they contained imaging artifacts. LCP belongs to the family Laemophloeidae, which is one of the smallest species of pantry beetles. They also have extremely thin elytra and faint patterns, which when imaged on filter papers (a common practice in food filth analysis), in some cases resulted in a fibrous paper background getting embedded in the elytral images (see Figure 6B). This imaging artifact was prominent in some parts of a few of the elytral images, which appeared quite different from the actual elytral pattern and could very well be the reason for their poorer performance. AAF was another species performing below average. In this case, each elytron had regions that appeared different from one another (variegated pattern). In some areas, the elytra appeared much brighter, while in other regions they appeared much darker. Some regions had more prominent patterns compared to others (see Figure 6C). When the images of the whole elytra were divided into subimages, the subimage set had much more pattern diversity. Some of the randomly-selected subimages used in testing probably appeared quite different from the training subimages, yielding a lower prediction value. It can also be noted that the AAF had a high Recall value but low Precision values. This indicated that our model was impressive in choosing relevant species, but in this case was slightly less exact due to highly diverse subimages.

While our collective results showed that model performance improved significantly when using better-quality images, thus validating our initial hypothesis, they indicated that species with higher intraspecies elytral diversity or with enhanced variegated elytral patterns do not perform as well. These observations seemed reasonable and have room for improvement without needing significant change in the model architecture. They are also aligned with a known general limitation of CNN models, which require training sets with both high-quality and large-quantity of images to yield better prediction accuracies (Valan et al., 2019; Høye et al., 2020).

While the cropped subimages were a way of imitating the actual beetle fragments and artificially increasing the size of the dataset, the limited number of elytral images remained one of the challenges in this study. Adding Keras image augmentation became a possible solution, as it has been used to solve imaging issues in domains such as medical image analysis (Shorten and Khoshgoftaar, 2019). In this step, several other scenarios, such as rotation, shearing, and zooming to some extent, were incorporated. During the training stage, the model was exposed to data augmentation to prepare it for possible variations, including likely presence of fragmented patterns. Even though this approach worked very well both for training accuracy and training loss, slightly lower accuracy and fluctuating loss observed in the validation stage also indicates that high variability of novel patterns is much harder to control and beyond the reach of data augmentation.

The broader objective of our work is to automate the process of elytral pattern recognition to better alleviate insect food contamination. We foresee this can only be achieved by concatenating the following three steps: (1) establishing a mechanism of acquiring high-quality images, (2) accumulating beetle images with proper labels in a repository with a growing number of samples for species with high variability, and (3) making them accessible for model development/improvement. Before developing a full-force effort to implement the whole process, it was critical to validate with a proof of concept the hypothesis that high-quality images can significantly improve predictive accuracy. The present study served this purpose and indicated that a high number of high-quality images is indeed a promising way forward in achieving precise identification over a large number of species. In our recent report on imaging optimization techniques, we elaborated on the method for acquiring high-quality images of pantry pests. Through this study, we developed a step-by-step procedure and a detailed instruction manual for high-quality image acquisition, which we will make publicly available. We currently are in the process of developing a high-quality image database containing 40 images per species for about 40 different pantry beetles, which will also be made public. Efforts currently are underway to construct a graphical user interface (GUI), from which any user can upload elytral images (preferably obtained by following the SOP and imaging manual) of pantry beetles in order to identify species using a CNN model similar to the one reported here. This use of the GUI will further enhance the high-quality image database and will provide a large number of high-quality, well-labeled image sets which can be used to further improve this CNN model in the future. At this point, the present work explores advantages and limitations of using a CNN model for classifying various species of pantry pests through elytral pattern recognition. We are optimistic that the current study has put us a step closer to achieving automated species identification of pantry pests, and thus toward a more efficient regulatory system to better manage food contamination scenarios.

## Conclusions

In this study, we aimed at scouring the landscape and moving closer to achieving near-perfect species-level identification. We set out to explore whether high-quality elytral images were sufficient for improving the prediction accuracy of pantry beetle species identification. To test this hypothesis, we first compared two CNN models; one developed with traditionally-obtained, low-resolution images, and another with optimized imaging conditions, yielding high-quality images. Overall, we observed an improvement in average prediction accuracy due to the improved image quality. When we extended the analysis to 27 different pantry beetles, we achieved an average accuracy of ~90%; however, several species fell below that average accuracy. A data review elucidated that below-average performance was not due to poor image quality, but rather to significant intraspecies variation of elytral pattern, and in some cases, to enhanced regional variation of patterns within one elytron. Detailed analysis indicated that greater numbers of high-quality images are necessary to account for these variations and achieve higher accuracy of the model. In future studies, we aim to achieve this objective using a publicly-available GUI for pantry beetle identification, allowing us to accumulate larger quantities of high-quality images through user participation. We hope this exploratory study will help achieve our ultimate goal of automated species identification of food-contaminating beetles.

## Data availability statement

The datasets presented in this study can be found in online repositories. The names of the repository/repositories and accession number(s) can be found below: https://github.com/hbisgin/BeetleCNN.

## Author contributions

TB acquired the images. HB performed the calculations. TB and HB analyzed the results with help from LW, NB, ZL, CF, and JX. HD and AB provided the in-house entomological support including samples and imaging facility. MP-R and JC provided the external entomological support and consultation. JX and HD led the projects. HV and WT managed and supported the study. All authors reviewed and approved the manuscript.

## Funding

This work was supported by NCTR Grant E0759101.

## Conflict of interest

Author CF was employed by company HK3 Lab. The remaining authors declare that the research was conducted in the absence of any commercial or financial relationships that could be construed as a potential conflict of interest.

## Publisher's note

All claims expressed in this article are solely those of the authors and do not necessarily represent those of their affiliated organizations, or those of the publisher, the editors and the reviewers. Any product that may be evaluated in this article, or claim that may be made by its manufacturer, is not guaranteed or endorsed by the publisher.

## Footnotes

^1^ https://github.com/hbisgin/beetleCNN
