# Supplementary figures and images for: Accurate species identification of food-contaminating beetles with quality-improved elytral images and deep learning
Source: Front Artif Intell. 2022 Aug 12;5:952424. doi: 10.3389/frai.2022.952424 (PMC9412741; doi:10.3389/frai.2022.952424)

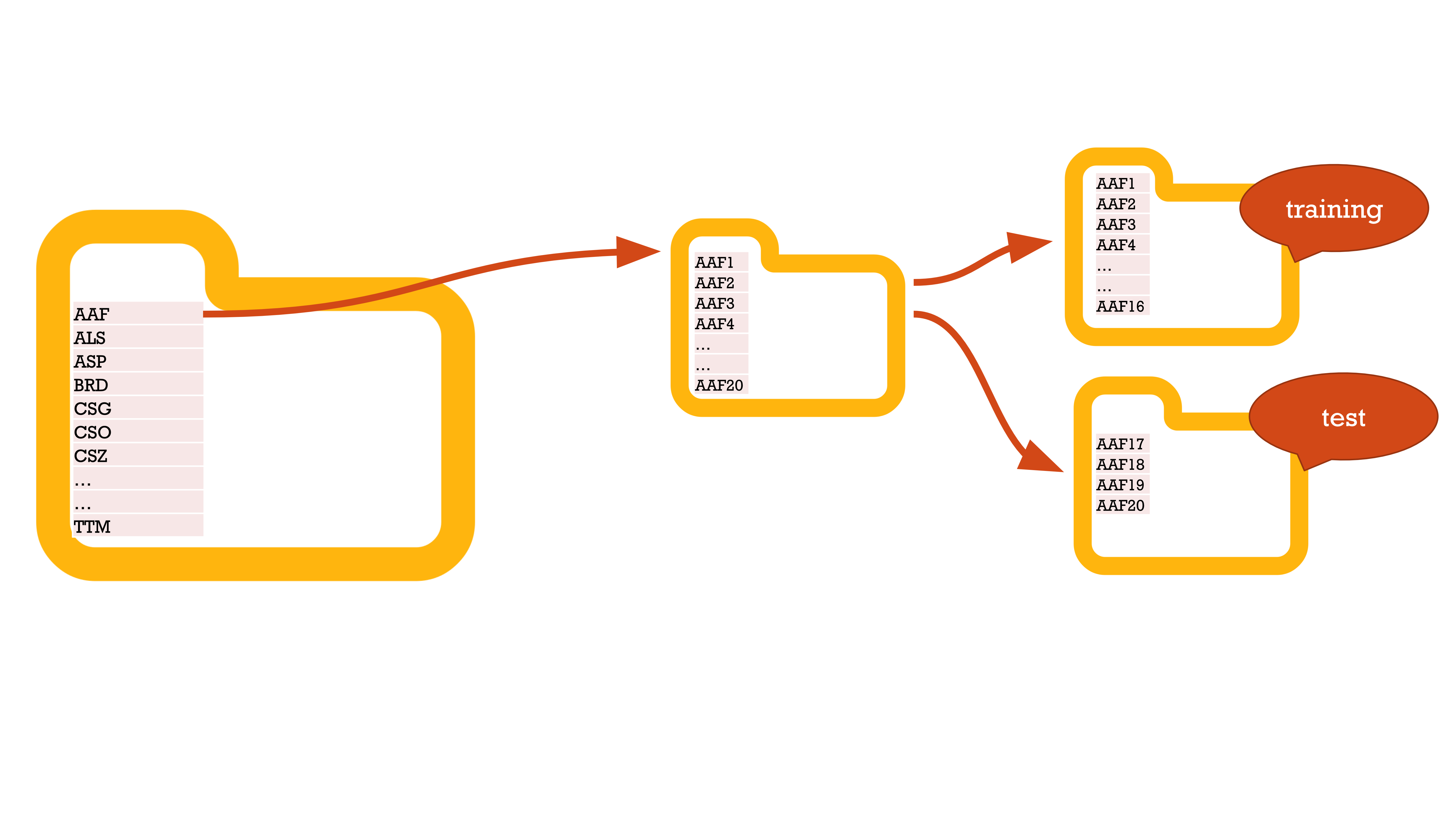

Supplement: Supplementary file 1 [file Image_1.JPEG]

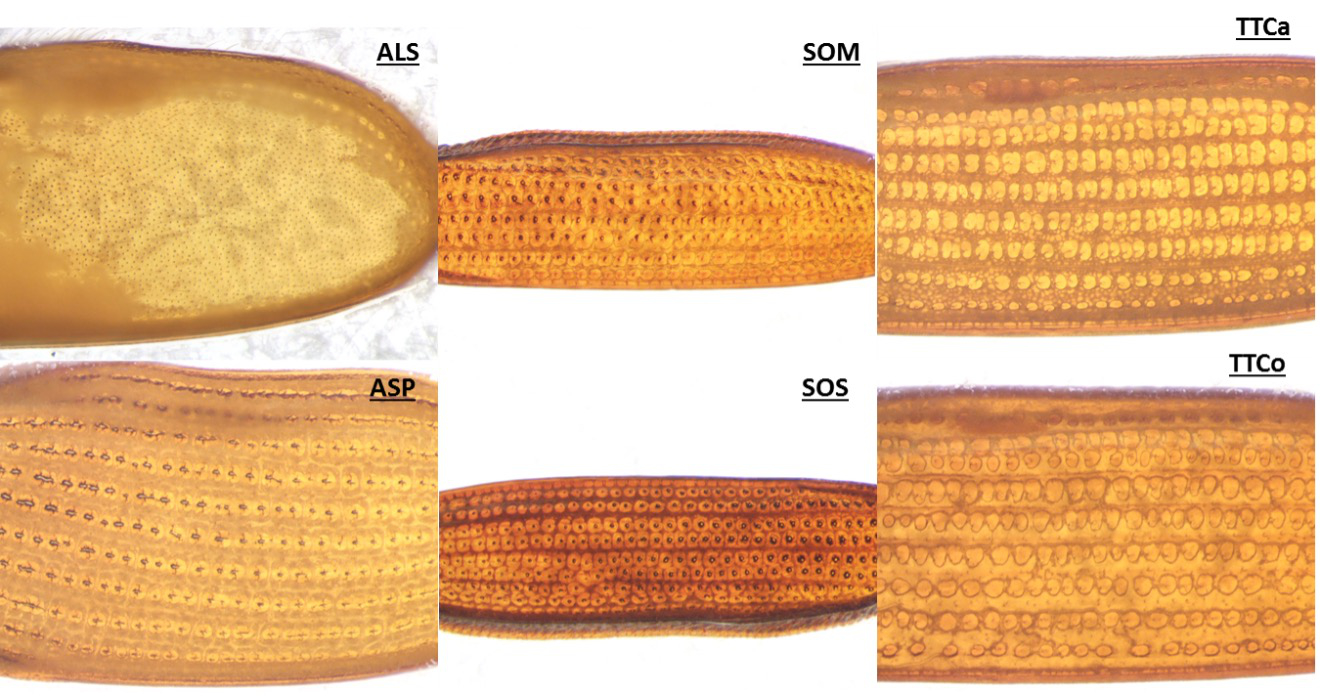

Supplement: Supplementary file 2 [file Image_2.JPEG]

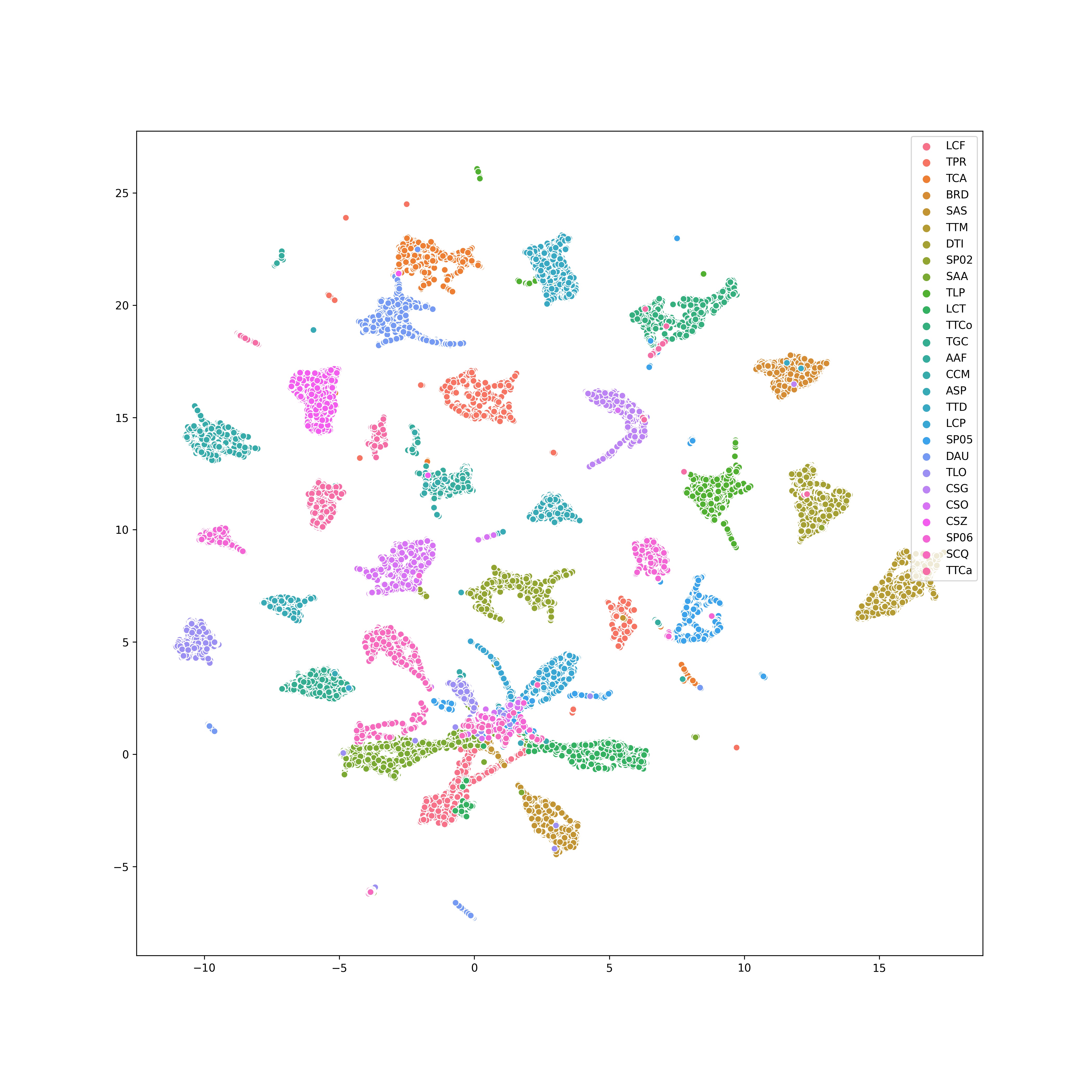

Supplement: Supplementary file 3 [file Image_3.JPEG]

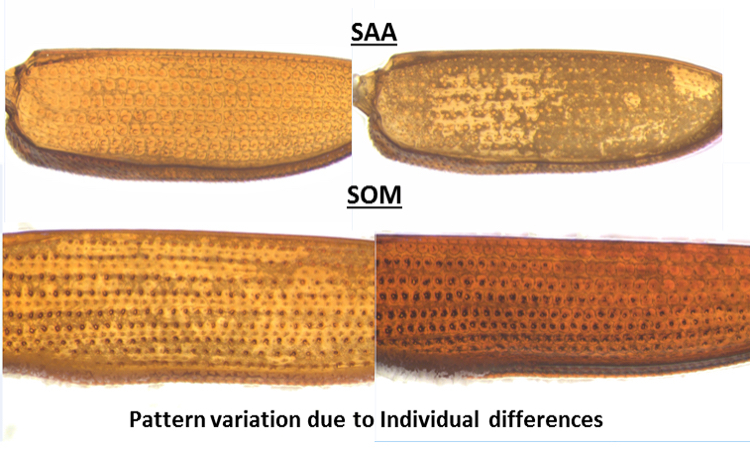

Supplement: Supplementary file 4 [file Image_4.JPEG]

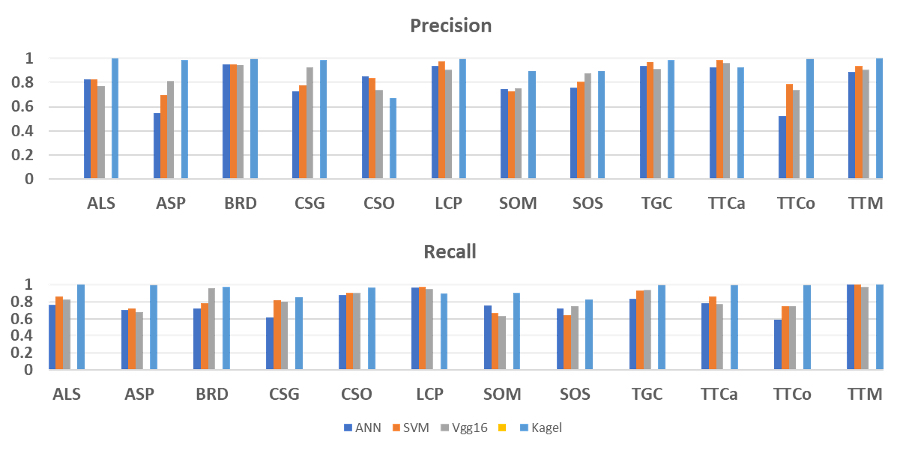

Supplement: Supplementary file 5 [file Image_5.JPEG]
